# Effects of quantitative feed restriction and sex on carcass traits, meat quality and meat lipid profile of Morada Nova lambs

**DOI:** 10.1186/s40104-017-0175-3

**Published:** 2017-05-22

**Authors:** Thiago L. A. C. de Araújo, Elzânia S. Pereira, Ivone Y. Mizubuti, Ana C. N. Campos, Marília W. F. Pereira, Eduardo L. Heinzen, Hilton C. R. Magalhães, Leilson R. Bezerra, Luciano P da Silva, Ronaldo L. Oliveira

**Affiliations:** 1 Department of Animal Science, Federal University of Ceara, Fortaleza, 60356001 Ceara Brazil; 2 Department of Animal Science, State University of Londrina, Londrina, 86051990 Paraná Brazil; 3 Laboratory of Sensory Analysis, Agency for Agricultural Research (EMBRAPA - Tropical Agroindustry), Fortaleza, 60511110 Ceará Brazil; 4 Department of Animal Science, Campus Professora Cinobelina Elvas, Federal University of Piauí, Bom Jesus, 64900000 Piaui Brazil; 5 Department of Animal Science, School of Veterinary Medicine and Animal Science/Federal University of Bahia, Salvador City, Bahia State 40.170-110 Brazil

**Keywords:** Dietary restriction, Fatty acid, Hair sheep, Lean meat, Semi-arid condition

## Abstract

**Background:**

An experiment was conducted to evaluate the effects of feed restriction (FR) and sex on the quantitative and qualitative carcass traits of Morada Nova lambs. Thirty-five animals with an initial body weight of 14.5 ± 0.89 kg and age of 120 d were used in a completely randomized study with a 3 × 3 factorial scheme consisting of three sexes (11 entire males, 12 castrated males and 12 females) and three levels of feeding (ad libitum – AL and 30% and 60% FR).

**Results:**

Entire males presented greater hot and cold carcass weights (*P* < 0.05), followed by castrated males and females. However, the hot carcass yield was higher for females and castrated males than for entire males. Luminosity values were influenced (*P* < 0.05) by sex, with entire males presenting higher values than castrated males and females. Females showed higher (*P* < 0.05) concentrations of linoleic acid and arachidonic acid in the meat of the *longissimus thoracis* muscle. The meat of animals submitted to AL intake and 30% FR showed similar (*P* > 0.05) concentrations, and the concentrations of palmitic acid, palmitoleic acid, stearic acid, oleic acid and conjugated linoleic acid were higher (*P* < 0.05) than those of animals with 60% FR. The meat of females had a higher ω6/ω3 ratio and lower h/H ratio, and females had greater levels of feeding. The meat of animals on the 60% FR diet had a greater ω6/ω3 ratio, lower h/H ratio and lower concentration of desirable fatty acids in addition to a greater atherogenicity index (AI) and thrombogenicity index (TI).

**Conclusion:**

Lambs of different sexes had carcasses with different quantitative traits without total influence on the chemical and physical meat characteristics. The lipid profile of the meat was less favorable to consumer health when the animals were female or submitted to 60% feed restriction.

## Background

Farming ruminants has an unquestionable importance to the economic and food security of many regions of the world, especially for tropical semi-arid regions [1], where sheep are highly relevant [2, 3]. In these regions, the environment interferes intensely with productive management strategies, and making decisions is crucial for the success of animal husbandry.

Production systems in semi-arid regions are based on the use of genetic resources with high adaptability and heat tolerance, which are heavily influenced by the qualitative and quantitative seasonality of food, but these local breeds are now threatened with extinction. Morada Nova is a prominent genetic group of hair sheep in semiarid regions of Brazil. These sheep are an indigenous group used for the production of meat and skin. They are smaller, have lower mortality rates, produce lighter carcasses, and are usually late to slaughter. Adult Morada Nova hair sheep weigh between 45 and 50 kg and can reproduce at approximately 8–9 mons of age with approximately 28 kg of body weight (BW) [4].

The improvement of animal yield by enhancing sustainable biodiversity may be a pathway toward greater food supplies. Such sustainable increases may be especially important for the 2 billion people reliant on small farms, many of which are undernourished, yet we know little about the efficacy of this approach.

Some productive strategies affect animal performance as well as the chemical and physical quality of the meat produced. The farmer can, for example, opt to obtain production rates and carcasses with different characteristics depending on the sex of the animals [5–7]. In tropical semi-arid regions, animals can be submitted naturally to periods of feed restriction (FR) due to feed supply variations or due to feed management planning, which is commonly used to save resources and reduce costs [8, 9]. Under these conditions, it may be assumed that in addition to lower performance, meat products may have different chemical and physical characteristics if the animals are sold [10, 11].

In the face of global concerns about the safety and nutritional quality of foods, it is necessary to understand the effects of commonly used production strategies not only on productivity but also on aspects related to human health, as is the case with lipid quality parameters. Thus, we simulate the impact of feed restriction in hair sheep of different sexes in semi-arid regions. We then evaluate their effects on carcass characteristics, meat quality and fatty acid profiles in the meat of Morada Nova lambs.

## Methods

### Animal care and location

This study was conducted at the Department of Animal Science, Federal University of Ceará, located in Fortaleza, CE, Brazil. Protocols (n° 98/2015) were in accordance with the standards established by the Committee of Ethics in Animal Research of the Federal University of Ceará.

### Animals, experimental design and management

Experimental lambs were obtained from the Morada Nova sheep breeding facility. The mating season was established with the objective of enabling a selection of animals with little variation in BW. Thirty-five lambs of the Morada Nova breed, including 23 males and 12 females, were selected. Twelve entire males were randomly assigned to the sexual class of castrated males, and the males were castrated using the burdizzo castrating method. Initially, the lambs had 14.5 ± 0.89 kg of BW and 120 d of age. The lambs were distributed in a completely randomized design in a 3 × 3 factorial scheme. Experimental treatments consisted of three sexes (11 entire males, 12 castrated males and 12 females) and three quantitative feeding levels (ad libitum (AL), 30% and 60% FR). The ration was formulated to supply the nutritional requirements of late maturity lambs with a gain of 150 g/d as recommended by the National Research Council (NRC) [12]. Before beginning data collection, the animals were randomly assigned to individual boxes provided with feed and water troughs, where they underwent an adaptive period of 15 d. Total mixed rations were provided twice a day (0730 and 1600 h), allowing for up to 10% orts only for animals fed AL. Before each morning feeding, the orts of each animal fed AL were removed and weighed to calculate the intake and feeding level of the lambs submitted to 30% and 60% FR (300 and 600 g/kg of FR). Thus, the restrictions were proportionally based on the intake of animals fed AL of each sex.

The ingredients used in the total ration and their proportions and composition are described in Table 1.

**Table 1:** Ingredient proportion and chemical composition of experimental rations

Ingredient	Proportion, % of DM			
Tifton 85 grass hay	60.0			
Ground corn grain	32.72			
Soybean meal	6.30			
Dicalcium phosphate	0.06			
Mineral premix ^a^	0.92			
Chemical composition, g/kg of DM	Total ration	Tifton 85 grass hay	Ground corn grain	Soybean meal
Dry matter	907.72	913.40	892.40	910.00
Crude protein	169.32	172.50	102.80	508.80
Ether extract	30.77	25.57	43.18	19.32
Ash	61.93	73.40	13.30	65.90
Neutral detergent fiber	438.65	668.20	112.54	134.63
NDFap ^b^	418.32	644.85	97.89	110.41
Acid detergent fiber	201.93	317.54	26.31	102.05
Non-fiber carbohydrate	319.67	58.30	728.19	273.30

^a^ Composition, 1 kg of premix: Calcium 225 g to 215 g; Phosphor 40 g; Sulfur 15 g; Sodium 50 g; Magnesium 10 g; Cobalt 11 mg; Iodine 34 mg; Manganese 1,800 mg; Selenium 10 mg; Zinc 2,000 mg; Iron 1,250 mg; Copper 120 mg; Fluor 400 mg; Vitamin A 37.5 mg; Vitamin D_3_ 0.5 mg and Vitamin E 800 mg

^b^ Neutral detergent fiber corrected for ash and protein

Samples of the roughage, concentrated and feed orts were taken to determine their chemical compositions and dry matter intake (DMI) of the lambs. The lambs were weighed every fifteen days to calculate BW gain (BWG). The trial period lasted 120 d. At the end of the trial period, the animals were weighed to determine total weight gain (TWG) and average daily gain (ADG).

### Slaughter, carcass data and meat samples

After 18 h of fasting, the animals were weighed to determine their BW at slaughter (BWS). The animals were then skinned and eviscerated according to the rules established in the Regulation of Brazilian Industrial and Sanitary Inspection of Animal Products. Subsequently, the lambs were stunned with the proper equipment, bled, skinned, and eviscerated. The viscera were weighed when filled, emptied, washed, drained and weighed when empty to determine the contents of the gastrointestinal tract and subsequently the empty BW (EBW) of the animals. The carcasses were identified and weighed to obtain the hot carcass weight (HCW) and yield (HCY) calculated in relation to BWS. After 24 h of cooling at 4 °C, the carcasses were weighed to obtain the cold carcass weight (CCW). Twenty-four hours *post mortem*, the pH was measured using a pH meter (HI-99163, Hanna® instruments, São Paulo, Brazil) by inserting the meter between the 4^th^ and 5^th^ lumbar vertebrae in the *longissimus lumborum* muscle.

The carcasses were sectioned with an electric saw (Ki Junta®, São Paulo, Brazil) along the spine, and the left halves of the carcasses were divided into six commercial cuts (leg, loin, ribs, lower ribs, neck and shoulder), which were individually weighed. A cross-sectional cut was made between the 12^th^ and 13^th^ ribs to expose the *longissimus thoracis (LT)* muscle, which measured the maximum distances between the ends of the muscle in the mediolateral direction (A) and dorsal-ventral (B) to subsequently calculate the rib eye area (REA) according to Eq. REA = (A / 2 × B / 2) × π. Subcutaneous fat thickness (SFT) was verified above measure B using a digital caliper. Samples were taken from the *LT* and *longissimus lumborum* (*LL*) muscles, vacuum packed and stored at −20 °C.

### Physicochemical meat analyses

The meat color was evaluated using a transverse cut on the back section, which was exposed to atmospheric air for 30 min before reading the oxygen myoglobin, which is the primary element that defines meat color [13]. As described by Miltenburg et al. [14], the coordinates L^*^, a^*^ and b^*^ were measured at three different points on the muscle, and the triplicates were averaged for each coordinate per animal. These measurements were performed using a Minolta CR-10 colorimeter (Konica® Minolta, Osaka, Japan) that was previously calibrated with the CIELAB system using a blank tile, illuminant D65 and 10° as the standard observation points. L^*^ is related to lightness (L^*^ = 0 black, 100 white); a^*^ (redness) ranges from green (−) to red (+); and b^*^(yellowness) ranges from blue (−) to yellow (+). Measurements were made from a 2° viewing angle using illuminant C. The color saturation (chroma, C^*^) was calculated as (a^*2^ + b^*2^)^1/2^ [15].

Meat samples of *LL* muscle were processed in a crusher to determine the water holding capacity (WHC), and cooking weight loss (CWL) was determined according to the American Meat Science Association (AMSA) [16] using *LL* meat samples (triplicate) without visible connective tissue that were previously thawed at 10 °C for 12 h. CWL indicated the difference in the weight of the meat before and after cooking on a preheated grill (George Foreman Jumbo Grill GBZ6BW, Rio de Janeiro, Brazil) at 170 °C. A digital skewer thermometer (Salcasterm 200®, São Paulo, Brazil) was used to monitor the internal temperature of the steak until the center reached 71 °C. Then, each steak was brought to room temperature, removed from the oven after temperature stabilization, and weighed again. The difference between the initial and final weights of a sample was used to determine the CWL, with the value expressed as a percentage.

After cooling at room temperature, the samples were again wrapped in foil and placed in a refrigerator (Consul CHB53C®, Salvador, Brazil) for 12 h at 4 °C. Fillets (5 ± 1) approximately 2 cm long, 1 cm wide and 1 cm high were cut from the meat to be evaluated for Warner-Bratzler shear force (WBSF). The instrumental texture analysis was performed on a TAXT2 texturometer (Stable Micro Systems Ltd., Vienna Court, UK) at 200 mm/min using standard shear blades (1.016 mm thick with a 3.05-mm blade). The instrumental texture analysis was performed according to the Research Center for Meat (US Meat Animal Research Center) and Shackelford et al. [17].

To evaluate lipid oxidation, meat samples of *LL* muscle stored under fast freezing at −20 °C for three months were thawed and crushed. Using the aqueous acid extraction method described by Cherian et al. [18], the 2-thiobarbituric acid reactive substances (TBARS) were measured in mg of malondialdehyde (MDA)/g of tissue.

Meat samples of *LT* muscle were evaluated for moisture, ash and protein contents, following method numbers 930.15, 920.153 and 928.08, respectively [19]. *LT* muscle samples were used to extract and quantify intramuscular fat (IMF). The fat of meat samples was isolated and purified using polar solvents (chloroform and methanol) according to the procedure of Folch et al. [20]. Aliquots of the fat extract were reserved and stored at −20 °C for subsequent use in determining the fatty acid profile.

### Fatty acid profile

To determine the fatty acid profile, the fat samples previously extracted from *LT* muscle were converted to fatty acid methyl esters (FAMEs). The FAMEs were prepared using a solution of methanol, ammonium chloride and sulfuric acid, following the procedure described by Hartman and Lago [21].

Samples were analyzed using a chromatograph (GC2010, Shimadzu®, São Paulo, Brazil) equipped with a flame-ionization detector and a biscyanopropyl polydimethylsiloxane capillary column of stationary phase (SP2560, 100 m × 0.25 mm, d_f_ 0.20 μm; Supelco®, Bellefonte, PA, USA). The column oven temperature was as follows: the initial temperature was held for 80 ° C, increased at 11 °C/min to 180 °C and at 5 °C/min to 220 °C and then maintained for 19 min. Hydrogen was used as a carrier gas at a flow rate of 1.5 mL/min, the split ratio was 1:30, and the injector and detector temperatures were 220 °C. The FAMEs were identified by a comparison of the FAME retention times with those of authentic standards (FAME mix components, Supelco®, Bellefont, PA, USA) following the same injection method. The results were quantified by normalizing the areas of the methyl esters and converted to mg/100 g of meat using a conversion factor of 0.92 for the contribution of fatty acids in lipids [22].

The concentrations of saturated fatty acids (SFAs), unsaturated fatty acids (UFAs), monounsaturated fatty acids (MUFAs), polyunsaturated fatty acids (PUFAs), ω6 and ω3 were calculated based on the fatty acid profile of the meat. Lipid quality indexes were determined using the sum of the desirable fatty acids [23], the thrombogenicity index (TI), the atherogenicity index (AI) [24], and the ratio between fatty acids hypocholesterolemic acid and hypercholesterolemic acid (h/H) [25]. The activity of enzymes involved in lipid metabolism, such as Δ9 desaturase in C16, Δ9 desaturase in C18 and elongase, were calculated according to the methods of Malau-Aduli et al. [26].

### Feed chemical analysis

To determine the chemical composition of the feed, triplicate samples were dried at 55 °C for 72 h in a forced-air oven, ground with a Willey mill (Tecnal®, São Paulo, Brazil) with a 1-mm sieve, and stored in airtight plastic containers (ASS®, São Paulo, Brazil). The samples were then stored in plastic jars with lids (ASS®, São Paulo, Brazil), labeled, and subjected to further laboratory analysis to measure the contents of dry matter (DM method 967.03), ash (method 942.05), crude protein (CP method 981.10), and ether extract (EE method 920.29) according to the Association of Official Analytical Chemists (AOAC) [27].

The neutral detergent fiber (NDF) content was determined as described by Van Soest et al. [28]. The acid detergent fiber (ADF) contents were determined as described by Robertson and Van Soest [29]. The NDF residue was incinerated in an oven at 600 °C for 4 h to determine the ash content, and the protein concentration was calculated by subtracting the neutral detergent insoluble protein (NDIP). NDF was corrected for the ash and protein contents. The Non-fiber carbohydrate (NFC) content was measured according to Mertens [30] and calculated based on the differences in the equation NFC = 100 – NDF – CP – EE – ash.

### Statistical analyses

Variables were subjected to analysis of variance using the GLM procedure of Statistical Analysis System - SAS® software [31] and the following equation: Y_ijk_ = μ + S_i_ + R_j_ + S_i_ × R_j_ + ε_ijk_, where Y_ijk_ is the dependent or response variable measured in the animal or experimental unit “k” of sexual class “i” at FR “j”; μ is the population mean or global constant; S_i_ is the effect of sexual class “i”; Rj is the effect of FR “j”; S_i_ × R_j_ is the interaction between effects of sexual class “i” and FR “j”; and ɛ_ijk_ is unobserved random error. Tukey-Kramer’s test was used to compare the means with a significance level of 5% probability (*P* < 0.05), and the same criterion was adopted for interactions between the effects of sex and FR.

## Results

### Performance and carcass traits

**Fig. 1:**
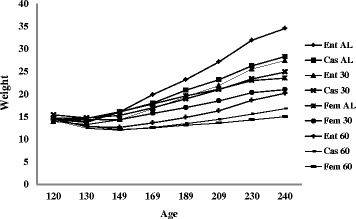
Evolution of animal weights during the experimental period. Average animal weight (kg) of each treatment in relation to approximate age in days from the beginning (120 d) to the end of the experiment (240 d); Ent AL = Entire males subjected to ad libitum intake; Cas AL = Castrated males subjected to ad libitum intake; Fem AL = Females subjected to ad libitum intake; Ent 30 = Entire males subjected to 30% feed restriction; Cas 30 = Castrated males subjected to 30% feed restriction; Fem 30 = Females subjected to 30% feed restriction; Ent 60 = Entire males subjected to 60% feed restriction; Cas 60 = Castrated males subjected to 60% feed restriction; Fem 60 = Females subjected to 60% feed restriction

There was an interaction (*P* < 0.05) between sex and FR for ADG, BWS and EBW (Table 2). In sum, females subjected to AL intake presented similar ADG, BWS and EBW (*P* > 0.05) to those of entire males and castrated males fed 30% FR (Table 3). Entire males fed AL presented higher (*P* < 0.05) ADG, BWS and EBW due to their higher growth (Table 3, Fig. 1).

**Table 2:** Performance of Morada Nova lambs of different sexes subjected to feed restrictions

Variables	Sexes			Feed restrictions			SEM	*P*-value		
	Ent	Cas	Fem	AL	30%	60%		Sex	Res	Sex × Res
IBW, kg	14.4	14.7	15.5	14.6	14.6	14.4	0.158	0.711	0.860	0.348
DMI, kg	0.66ª	0.60^b^	0.52^c^	0.80ª	0.62^b^	0.36^c^	0.009	<.0001	<.0001	0.418
BWS, kg	27.4ª	23.3^b^	19.9^c^	28.8^a^	24.4^b^	17.3^c^	0.308	<.0001	<.0001	0.047
EBW, kg	21.0ª	18.1^b^	16.1^c^	23.0ª	19.0^b^	13.3^c^	0.214	<.0001	<.0001	0.038
ADG, g	106ª	71.7^b^	42.5^c^	116ª	80.8^b^	23.3^c^	2.317	<.0001	<.0001	0.029

*Ent* Entire males, *Cas* Castrated males, *Fem* Females, *AL* ad libitum intake, *30%* 30% feed restriction, *60%* 60% feed restriction, *SEM* standard error of the mean, *Sex* sexes and Res = feed restriction, *IBW* initial body weight, *DMI* dry matter intake, *BWS* body weight at slaughter, *EBW* empty body weight, *TWG* total weight gain, *ADG* average daily gain

^a b c^ Means followed by different letters differ between sexes according to a Tukey-Kramer test (*P* < 0.05)

^a b c^ Means followed by different letters differ between feed restrictions according to a Tukey-Kramer test (*P* < 0.05)

**Table 3:** Interactions between sexes and feed restriction on performance of Morada Nova lambs

Variables	Entire male			Castrated male			Female		
	AL	30%	60%	AL	30%	60%	AL	30%	60%
BWS	35^adA^	27^bdBC^	20^cdEF^	28^aeB^	25^bdBCD^	17^ceFG^	23^afCDE^	21^bdeDEF^	15^bfG^
EBW	27 ^adA^	21^bdBC^	15^cdEF^	22^aeB^	19^beCD^	13^cdEF^	20^aeBCD^	17^bfDE^	12^ceG^
ADG	164^adA^	110^bdBC^	47^cdDE^	114^aeB^	78^beCD^	21^ceEF^	72^afD^	54^afD^	5^bfF^

*AL* ad libitum intake, *30%* 30% feeding restriction, *60%* 60% feeding restriction. *BWS* body weight at slaughter (kg), *EBW* empty body weight (kg), *ADG* average daily gain (g)

^abc^; Means followed by different letters in same sexes differ by Tukey-Kramer test (*P* < 0.05)

^def^; Means followed by different letters in same feeding restriction level differ by Tukey-Kramer test (*P* < 0.05)

^ABCDEFG^; Means followed by different capital letters in same line differ by Tukey-Kramer test (*P* < 0.05)

Except for SFT, the carcass traits that were analyzed (HCW, HCY, CCW, CCY and REA) were influenced (*P* < 0.05) by sex (Table 4). Entire males showed higher means of HCW and CCW followed by castrated males and females. However, females and castrated males had a higher HCY than did entire males. After cooling and considering the losses caused by this process, only females had the highest yield (CCY), whereas castrated males did not differ from the other sexes. The level of FR did not influence (*P* > 0.05) the HCY and CCY, which indicated that the lower weights due to lower feed intake occurred proportionately throughout the bodies of the animals. HCW, CCW and SFT decreased with increasing FR (60%).

There was an interaction (*P* < 0.05) between sex and FR in neck weight (Table 4). However, no clear result was evidenced (Table 5). The weights of all commercial cuts were influenced (*P* < 0.05) by sex and by FR (Table 4). Entire males had heavier cuts, followed by castrated males and females, which reflected the effects observed with the CCW. The weights of the commercial cuts decreased due to the reduction in feeding supply.

**Table 4:** Carcass characteristics and commercial cuts weight of Morada Nova lambs of different sexes subjected to feed restriction

Variables	Sexes			Feed restrictions			SEM	*P*-value		
	Ent	Cas	Fem	AL	30%	60%		Sex	Res	Sex × Res
HCW, kg	11.5ª	10.3^b^	8.99^c^	12.7ª	10.6^b^	7.51^c^	0.139	<.0001	<.0001	0.110
HCY, %	42.1^b^	44.1ª	45.2ª	44.4	43.6	43.4	0.325	0.002	0.474	0.967
CCW, kg	11.4ª	10.18^b^	8.88^c^	12.58ª	10.51^b^	7.42^c^	0.138	<.0001	<.0001	0.106
CCY, %	41.7^b^	43.6^ab^	44.7ª	43.9	43.2	42.9	0.315	0.002	0.435	0.965
SFT, mm	1.32	1.10	1.12	1.55^a^	1.25ª	0.75^b^	0.060	0.283	<.0001	0.185
REA, cm^2^	11.0ª	10.3^ab^	9.36^b^	11.7ª	10.1^b^	8.88^b^	0.205	0.010	<.0001	0.649
Leg, kg	1.84^a^	1.64^b^	1.48^c^	2.02^a^	1.69^b^	1.26^c^	0.022	<.0001	<.0001	0.109
Loin, kg	0.58^a^	0.48^b^	0.42^b^	0.65^a^	0.50^b^	0.32^c^	0.011	<.0001	<.0001	0.102
Neck, kg	0.40^a^	0.35^b^	0.27^c^	0.40^a^	0.33^b^	0.28^c^	0.007	<.0001	<.0001	0.010
Shoulder, kg	1.01^a^	0.93^b^	0.83^c^	1.12^a^	0.93^b^	0.70^c^	0.011	<.0001	<.0001	0.383
Rib, kg	1.07^a^	0.86^b^	0.78^b^	1.11^a^	0.98^a^	0.62^b^	0.024	0.0002	<.0001	0.329
Lower rib, kg	0.85^a^	0.79^a^	0.63^b^	0.96^a^	0.80^b^	0.51^c^	0.019	0.0002	<.0001	0.485

*Ent* Entire males, *Cas* Castrated males, *Fem* Females, *AL* ad libitum intake, *30%* 30% feed restriction, *60%* 60% feed restriction, *SEM* standard error of the mean, *Sex* sexes and Res = feed restriction, *HCW* hot carcass weight, *HCY* Hot carcass yield, *CCW* cold carcass weight, *CCY* cold carcass yield, *SFT* subcutaneous fat thickness, *REA* Rib eye area

^a b c^ Means followed by different letters differ between sexes according to a Tukey-Kramer test (*P* < 0.05)

^a b c^ Means followed by different letters differ between feed restrictions according to a Tukey-Kramer test (*P* < 0.05)

**Table 5:** Interactions between sexes and feed restriction on the weight of commercial cuts of Morada Nova lambs

Variables	Entire male			Castrated male			Female		
	AL	30%	60%	AL	30%	60%	AL	30%	60%
Neck	0.51^adA^	0.36^bBC^	0.33^bdBC^	0.42^adAB^	0.33^bBC^	0.29^bdCD^	0.28^abeCD^	0.32^aBCD^	0.21^beD^

*AL* ad libitum intake, *30%* 30% feeding restriction, *60%* 60% feeding restriction

^abc^; Means followed by different letters in same sexes differ by a Tukey-Kramer’s test (*P* < 0.05)

^def^; Means followed by different letters in same feeding restriction level differ by a Tukey-Kramer test (*P* < 0.05)

^ABCD^; Means followed by different capital letters in same line differ by a Tukey-Kramer test (*P* < 0.05)

### Physicochemical meat quality

L* was influenced (*P* < 0.05) by sex (Table 6). The color parameters a*, b* and C* were not affected by sex or FR. Castrated males and females did not differ (*P* > 0.05), and they had lower values (*P* < 0.05) than did entire males. Animals subjected to 60% FR showed a higher 24 h *post-mortem* pH in their meat compared to that of animals subjected to AL intake.

**Table 6:** Physicochemical quality of Morada Nova lamb meat of different sexes subjected to feed restriction

Variables	Sexes			Feed restrictions			SEM	*P*-value		
	Ent	Cas	Fem	AL	30%	60%		Sex	Res	Sex × Res
L*	39.7ª	38.2^b^	37.5^b^	38.2	38.4	38.8	0.244	0.003	0.657	0.659
a*	22.2	21.8	21.9	21.9	22.3	21.7	0.233	0.767	0.541	0.689
b*	9.83	9.43	8.80	9.36	9.93	8.77	0.220	0.197	0.126	0.866
C*	24.3	23.8	23.6	23.8	24.4	23.4	0.320	0.386	0.252	0.771
WHC, %	35.9	34.5	35.0	34.7	32.8	37.9	1.045	0.859	0.154	0.511
CWL, %	38.6	38.5	37.4	37.4	38.5	38.6	0.403	0.443	0.408	0.937
pH	5.61	5.60	5.66	5.57^b^	5.62^ab^	5.69ª	0.019	0.354	0.044	0.667
Shear force, N	42.9	46.1	39.2	44.0	41.4	42.8	1.623	0.236	0.808	0.702
TBARS	0.72	0.75	0.85	0.82	0.64	0.86	0.041	0.395	0.073	0.534
Moisture, %	76.5	75.9	75.8	75.4^b^	75.9ª	76.9ª	0.146	0.167	0.001	0.064
Protein, %	18.6	18.9	19.0	18.9	18.5	19.1	0.117	0.332	0.139	0.018
Fat, %	3.99	4.32	4.18	4.74ª	4.70ª	3.05^b^	0.107	0.468	<.0001	0.680
Ash, %	0.92	0.90	0.93	0.92^b^	0.87^b^	0.98^a^	0.009	0.327	<.0001	0.155

*Ent* Entire males, *Cas* Castrated males, *Fem* Females, *AL* ad libitum intake, *30%* 30% feed restriction, *60%* 60% feed restriction, *SEM* standard error of the mean, *Sex* sexes and Res = feed restriction, Color parameter L* lightness, 0 black and 100 white, Color parameter a* redness, ranges from green (−) to red (+), Color parameter b* yellowness, ranges from blue (−) to yellow (+), Color parameter C* chroma; C* = (a^*2^ + b^*2^)^1/2^(MacDougall and Taylor, 1975), *WHC* water holding capacity, *CWL* Cooking weight losses, *TBARS* 2-thiobarbituric acid reactive substance, *MDA* malondialdehyde

^a b^ Means followed by different letters differ between sexes according to a Tukey-Kramer test (*P* < 0.05)

^a b^ Means followed by different letters differ between feed restrictions according to a Tukey-Kramer test (*P* < 0.05)

There was no effect (*P* > 0.05) of FR and sex on the protein content in meat from *LT* muscle (Table 6). However, an interaction (*P* < 0.05) between sex and FR for this variable was observed. Females fed AL had a higher (*P* < 0.05) protein content than did females submitted to 30% FR (Table 7). The moisture content in the *LT* muscle of animals with AL intake was lower (*P* < 0.05) compared to that of animals subjected to 30 and 60% FR (Table 6). Animals submitted to AL intake and 30% FR provided similar (*P* > 0.05) amounts of IMF, whereas animals subjected to 60% FR had a reduced (*P* < 0.05) concentration of IMF. Ash percentage was higher (*P* < 0.05) in the meat from animals subjected to 60% FR.

**Table 7:** Interactions between sexes and feed restriction on Meat composition of Morada Nova lambs

Variables	Entire male			Castrated male			Female		
	AL	30%	60%	AL	30%	60%	AL	30%	60%
Protein	18.24^AB^	18.70^AB^	18.89^AB^	18.57^AB^	18.66^AB^	19.46^AB^	19.99^A^	18.22^B^	18.93^AB^

*AL* ad libitum intake, *30%* 30% feeding restriction, *60%* 60% feeding restriction

^abc^; Means followed by different letters in same sex differ by a Tukey-Kramer test (*P* < 0.05)

^def^; Means followed by different letters in same feeding restriction level differ by a Tukey-Kramer test (*P* < 0.05)

^AB^; Means followed by different capital letters in same line differ by a Tukey-Kramer test (*P* < 0.05)

### Fatty acid profile

It was not possible to separate the peaks of conjugated linoleic acid (CLA) isomers normally identified in meats from ruminants. Thus, the nomenclature used covered all isomers (CLA). There was an interaction (*P* < 0.05) between sex and FR for elaidic acid (C18:1 t9) and behenic acid (C22:0) in the meat of the *LT* muscle (Table 8). However, after adjusting for multiple comparisons, a clear interaction response was only detected for elaidic acid (Table 9). Probably data set characteristics contributed to the absence of significance after the Tukey-Kramer test, contradicting the initial result of the ANOVA for the behenic acid. Females submitted to 60% FR had higher amounts of elaidic acid than did entire males and castrated males submitted to 60% FR (Table 9).

**Table 8:** Fatty acids profile in the meat of the *longissimus thoracis* muscle of Morada Nova lambs of different sexes subjected to feed restriction

Fatty acids, mg/100 g of meat	Sexes			Feed restrictions			SEM	*P*-value		
	Ent	Cas	Fem	AL	30%	60%		Sex	Res	Sex × Res
C10:0	4.70	6.56	6.05	7.18	5.24	4.89	0.700	0.540	0.392	0.913
C12:0	32.8	59.7	43.6	44.9	35.1	56.1	6.476	0.254	0.432	0.648
C14:0	87.3	89.8	108	96.0^ab^	110^a^	78.5^b^	4.568	0.156	0.027	0.937
C14:1c9	8.51	10.3	9.95	10.9	9.64	8.26	0.610	0.447	0.255	0.436
C15:0	22.6	29.3	33.0	23.6	26.72	34.6	1.984	0.122	0.084	0.572
C16:0	912	914	966	1069^a^	1080^a^	642^b^	30.20	0.711	<.0001	0.816
C16:1c9	41.2	40.3	43.8	49.0^a^	48.0^a^	18.3^b^	1.660	0.685	<.0001	0.551
C17:0	44.4	47.2	48.6	48.9	46. 9	44.4	1.047	0.277	0.245	0.314
C17:1c10	18.9	16.8	17.3	18.9	17.37	16.8	0.901	0.635	0.606	0.300
C18:0	620	687	649	695^a^	701^a^	561^b^	19.03	0.377	0.008	0.770
C18:1c9	1474	1639	1474	186^a^	1814^a^	911^b^	41.65	0.192	<.0001	0.604
C18:1 t9	36.0	36.1	44.5	41.1	38.4	37.1	1.819	0.106	0.666	0.032
C18:2c9c12	70.1^b^	83.1^ab^	91.4^a^	82.8	79.1	82.8	2.388	0.005	0.768	0.582
CLA	3.96	4.92	4.54	5.29^a^	4.99^a^	3.14^b^	0.289	0.410	0.016	0.397
C18:3c9c12c15	18.5	21.6	21.0	22.2	20.5	18.4	0.701	0.201	0.109	0.510
C20:4c5c8c11c14	31.2^b^	37.3^ab^	39.2^a^	33.0	33.2^b^	41.6^a^	1.236	0.045	0.010	0.862
C20:5c5c8c11c14c17	7.57	8.73	8.75	7.74^b^	7.39^b^	9.92^a^	0.216	0.061	<.0001	0.171
C22:0	8.08	9.13	9.02	8.24	8.51	9.48	0.253	0.201	0.143	0.042
Unidentified	550	583	565	618	617	474	-	-	-	-

*Ent* Entire males, *Cas* Castrated males, *Fem* Females, *AL* ad libitum intake, *30%* 30% feed restriction, *60%* 60% feed restriction, *SEM* standard error of the mean, *Sex* sexes and Res = feed restriction, *C10:0* Capric acid, *C12:0* Lauric acid, *C14:0* Myristic acid, *C14:1c9* Myristoleic acid, *C15:0* Pentadecylic acid, *C16:0* Palmitic acid, *C16:1c9* Palmitoleic acid, *C17:0* Margaric acid, *C17:1c10* Heptadecenoic acid, *C18:0* stearic acid, *C18:1c9* Oleic acid, *C18:1 t9* Elaidic acid, *C18:2c9c12* Linoleic acid, *C18:3c9c12c15* α-Linolenic acid, *C20:4c5c8c11c14* Arachidonic acid, *C20:5c5c8c11c14c17* Eicosapentaenoic acid, *C22:0* Behenic acid, *CLA* Conjugated linoleic acids: rumenic acid and their isomers

^a b^ Means followed by different letters differ between feed restrictions according to a Tukey-Kramer test (*P* < 0.05)

**Table 9:** Interactions between sexes and feed restriction on fatty acids profile in the meat of the *longissimus thoracis* muscle of Morada Nova lambs

Fatty acids, mg/100 g of meat	Entire male			Castrated male			Female		
	AL	30%	60%	AL	30%	60%	AL	30%	60%
C18:1 t9	38.6^AB^	41.8^AB^	28.6^B^	45.5^AB^	34.6^AB^	28.1^B^	39.2^AB^	39.9^AB^	54.5^A^
C22:0	8.2	8.6	7.4	9.1	8.0	10.2	7.3	8.9	10.8

*AL* ad libitum intake, *30%* 30% feeding restriction, *60%* 60% feeding restriction

^AB^; Means followed by different capital letters in same line differ by Tukey-Kramer test (*P* < 0.05)

There was an effect (*P* < 0.05) of sex only on the concentrations of linoleic acid (C18:2 c9c12) and arachidonic acid (C20:4 c5c8c11c14) (Table 8). Females showed higher (*P* < 0.05) concentrations of these fatty acids than did entire males and castrated males, which showed no difference (*P* > 0.05) compared to the other categories. Meat of animals submitted to AL intake and 30% FR showed similar (*P* > 0.05) and higher (*P* < 0.05) values compared to those of animals subjected to 60% FR for concentrations of palmitic acid (C16:0), palmitoleic acid (C16:1c9), stearic acid (C18:0), oleic acid (C18:1c9) and CLA. The concentration of myristic acid (C14:0) was higher (*P* < 0.05) in meat from animals subjected to 30% FR than in meat from animals subjected to 60% FR. Meat from animals subjected to 60% FR showed greater (*P* < 0.05) concentrations of arachidonic acid and eicosapentaenoic acid (C20:5c5c8c11c14c17 - EPA) than did meat from animals subjected to AL intake and 30% FR.

The concentration of PUFAs was greater in the meat of females and lower in the meat of entire males (Table 10). However, the meat of females provided a higher ω6/ω3 ratio and AI in addition to presenting a lower h/H ratio. The activity of the elongase enzyme was higher in the *LT* muscle of castrated males. The sum of SFA, UFA and PUFA was similar (*P* > 0.05) in the meat of animals subjected to AL intake and 30% FR and was higher (*P* < 0.05) compared to that in animals subjected to 60% FR. The meat of animals subjected to 60% FR provided a greater ω6/ω3 ratio, lower h/H ratio and lower concentration of desirable fatty acids, in addition to a greater AI and TI.

**Table 10:** Fatty acid classes, ratios, indexes and enzyme activity in meat of l*ongissimus thoracis* muscle of Morada Nova lambs of different sexes subjected to feed restriction

Index, mg/100 g of meat	Sexes			Feed restrictions			SEM	*P*-value		
	Ent	Cas	Fem	AL	30%	60%		Sex	Res	Sex × Res
ΣSFA	1731	1837	1853	1988^a^	2007^a^	1425^b^	50.12	0.579	<.0001	0.770
ΣUFA	1707	1896	1748	2131^a^	2069^a^	1151^b^	44.52	0.210	<.0001	0.602
ΣMUFA	1578	1741	1585	1981^a^	1926^a^	997^b^	44.10	0.248	<.0001	0.569
ΣPUFA	129^b^	155^ab^	163^a^	150	142	154	4.222	0.008	0.504	0.716
Σω6	99.0^b^	120^a^	131^a^	116	110	124	3.338	0.003	0.212	0.777
Σω3	26.10	30.3	28.8	29.9	27.9	27.4	0.909	0.188	0.515	0.628
ω6/ω3	3.84^b^	3.99^b^	4.53^a^	3.90^b^	3.96^b^	4.50^a^	0.076	0.003	0.009	0.784
h/H	1.60^ab^	1.77^a^	1.54^b^	1.74^a^	1.65^ab^	1.52^b^	0.027	0.008	0.015	0.661
DFA	2328	2583	2397	2826^a^	2769^a^	1713^b^	56.65	0.185	<.0001	0.661
TI	1.78	1.70	1.81	1.64^b^	1.72^b^	1.93^a^	0.031	0.351	0.003	0.741
AI	0.75^b^	0.72^b^	0.84^a^	0.70^b^	0.74^b^	0.87^a^	0.016	0.020	<.0001	0.706
Δ^9^ desaturase C16	4.26	4.17	4.28	4.36	4.28	4.07	0.124	0.925	0.631	0.872
Δ^9^ desaturase C18	69.8	69.0	67.9	72.6^a^	72.1^a^	61.9^b^	0.556	0.428	<.0001	0.431
Elongase	68.9^b^	70.9^a^	67.4^b^	69.6	69.1	68.4	0.273	<.0001	0.240	0.179

*Ent* Entire males, *Cas* Castrated males, *Fem* Females, *AL* ad libitum intake, *30%* 30% feed restriction, *60%* 60% feed restriction, *SEM* standard error of the mean, *Sex* sexes and Res = feed restriction

*SFA* saturated fatty acids (ΣC10:0, C12:0, C14:0, C15:0, C16:0, C17:0, C18:0, C22:0); *UFA* unsaturated fatty acid (ΣC14:1c9, C16:1c9, C17:1c9, C18:1c9, C18:1 t9, C18:2c9c12, C18:2t9t12, C18:2c9t11, C18:3c6c9c12, C20:4c5c8c11c14, C20:5c5c8c11c14c17), *MUFA* monounsaturated fatty acid (ΣC14:1c9, C16:1c9, C17:1c9, C18:1c9, C18:1 t9), *PUFA* polyunsaturated fatty acid (ΣC18:2c9c12, C18:2t9t12, C18:2c9t11, C18:3c6c9c12, C20:4c5c8c11c14, C20:5c5c8c11c14c17), *ω6* (ΣC18:2c9c12, C18:2t9t12, C20:4c5c8c11c14), *ω3* (C18:3c9c12c15, C20:5c5c8c11c14c17), *ω6:ω3* relation (Σω6/Σω3), *h/H* hypocholesterolemic hypercholesterolemic relation (ΣC18:1c9, C18:2c9c12, C18:3c6c9c12, C20:4c5c8c11c14, C20:5c5c8c11c14c17 / ΣC14:0, C16:0), *DFA* Desirable fatty acids (ΣMUFA, PUFA, C18:0), *TI* Thrombogenicity index [(ΣC14:0, C16:0, C18:0) / Σ(0,5xΣMUFA), (0,5xΣω6), (3xΣω3), (Σω3/Σω6)], *AI* Atherogenicity index {[ΣC12:0,(4xC14:0), C16:0] / [(Σω3,ω6) + (C18:1c9) + (Σ other MUFA)]}; Enzyme activity Δ^9^ desaturase on fatty acid with 16 carbons {100[(C16:1c1) / (ΣC16:1c1, C16:0)]}; Enzyme activity Δ^9^ desaturase on fatty acid with 18 carbons {100[(C18:1c1) / (ΣC18:1c1, C18:0)]}; Elongase activity enzyme {100[(ΣC18:0,C18:1c9) / (ΣC16:0,C16:1c9,C18:0,C18:1c9)]}

^a b^ Means followed by different letters differ between sexes according to a Tukey-Kramer test (*P* < 0.05)

^a b^ Means followed by different letters differ between feed restrictions according to a Tukey-Kramer test (*P* < 0.05)

## Discussion

### Performance, carcass traits and physicochemical meat quality

Productions systems in semi-arid regions are based on the use of genetic resources with high adaptability and heat tolerance, which are heavily influenced by qualitative and quantitative seasonality of food [32, 33]. An example is the Morada Nova, an important indigenous breed of hair sheep in northeastern Brazil that is used for meat and skin production and is highly valued on the international market [34]. Weight loss has a strong impact on animal productivity [35], compromising the animal welfare and income of farmers worldwide [33]. In our study, we observed an absence of growth in females with 60% FR and a low ADG in lambs with 30% FR. This effect is a response to a lower amount of nutrients [36] because lambs have higher energy and protein requirements for growth [32, 37], which demand higher intakes. The study of van Harten et al. [38] showed that in animals under FR, lipids are mobilized and transformed into energy. This occurs to meet the net energy requirements for maintenance. In addition to genetic and nutritional factors, sex is variable and impacts animal productive responses.

Effects of interactions between sex and FR showed that females performed similarly to entire males and castrated males with 30% FR. Such effects reflect the empty cup weight gain, when females generally present lower rates of protein deposition in the empty body [39].

A sex effect is evident in the regulation of adiposity and muscularity and has been attributed to sexual steroid hormones [40] because testosterone promotes an increase in body mass [41]. These effects were observed in this study, in which entire males had higher carcass weights and commercial cuts. Castrated males [6] and females [42] present higher HCY attributed to increased fat deposit during weight gain [43]. Furthermore, the higher central or intra-abdominal accumulation of fat in male individuals [44] contributed to greater proportions of non-carcass components and consequently had a lower carcass yield.

Dietary restrictions have reduced the accumulation of the body stores of fat and protein, which resulted in lighter carcasses and commercial cuts. Nutritional limitations reduce cell proliferation and differentiation in tissues in response to reduced local production of insulin-like growth factor-1 (IGF-1), as signaled by the state of relative resistance of growth hormone (GH) [45]. In addition, in the post-absorptive state, non-esterified fatty acids, glycerol, alanine and glycine are oxidized, which supplies part of the energy demand [46]. Thus, in situations of lower nutritional intake, some of the body energy reserves are consumed. This situation was observed in animals submitted to the levels of FR used in this research.

The 24 h *post-mortem* pH was higher in the meat from animals subjected to 60% FR, which may be related to the lower content of muscle glycogen caused by lower feed intake and intense mobilization of reserves during the development of these animals. However, the pH remained between 5.5 and 5.8, which is desirable for meat [47]. An increase in the pH of meat can increase the activity of cytochrome oxidase by reducing the uptake of oxygen by myoglobin, which results in a purplish red color [48]. However, the higher pH in meat from animals subjected to 60% FR was not enough to influence the color and other quality parameters analyzed in this study.

L* was greatly influenced by the pigment contents, especially those of hematin, myoglobin and their forms [49]. Chromophores, such as myoglobin and hemoglobin, absorb visible light by increasing light penetration and consequently decreasing reflectance [50]. Sañudo et al. [51] observed more myoglobin in females (2.90 mg/g of meat) than in entire males (2.56 mg/g of meat) and reported a similar effect of sex on L*, recording 39.80% for females and 41.26% for entire males. More myoglobin may explain the color variation in the meat of female animals observed in the present study.

Testosterone, besides providing an anabolic effect [41], acts on plasma glucose levels in males but does not alter the phosphorylation of AMP-activated protein kinase in muscle [40], which influences the concentration of IMF in males to a small degree. A similar result was observed in this study because the percentage of meat fat was similar between the sexes and did not reflect the effects of higher HCW in entire males.

Variations in meat fat concentration occur mainly due to changes in balance between dietary energy and nutrient requirements [52]; thus, the least amount of energy consumed by animals subjected to 60% FR decreased the deposition of IMF. FR of 60% meets the net energy needs for maintenance and obviously does not prioritize nutrients for the deposition of adipose tissue. However, 30% FR did not cause significant changes to this balance or to the concentration of IMF in *LT* muscle.

### Fatty acid profiles

Studies on cattle have indicated increased incorporation of long chain fatty acids into the phospholipids of heifer meat in response to concentrations of plasmalogens [53]. The effects observed on the concentration of linoleic and arachidonic acids in meat of females indicate that this incorporation can also occur in lamb meat but was not observed due to the need for a more detailed analysis. The effect of the interaction between sex and FR on elaidic acid concentration shows that under 60% FR, females accumulate a greater amount of this acid. It becomes important to know the unique physiological functions of specific isomers as well as their origin. A portion of the trans-11 C18:1 isomer produced by ruminal microbes is converted into cis-9, trans-11 C18: 2 by tissue desaturase [54]; however, this cannot occur with elaidic acid (C18:1 t9). The quality lipid indexes showed that the meat of female animals had a lipid profile with less desirable characteristics compared to that of meat from other sexes due to a higher AI and a lower h/H ratio. The higher ω6/ω3 ratio would also indicate lower quality meat fat in females [55]; however, the latest recommendations suggest no rational limit for this ratio if the intake of ω6 and ω3 is within the proper range for human diets [56].

More feeding can explain the higher concentration of palmitic acid, palmitoleic acid, stearic acid and oleic acid in the meat of animals subjected to AL intake and 30% FR. These effects reflect the results observed with the IMF content. As 30% FR did not significantly influence the deposition of IMF, there was no effect on the composition of fatty acids. The kinetics of the feed in the rumen may also have influenced the exposure time of the fatty acids to biohydrogenation [57]. The more severe restriction may have resulted in a lower passage rate, higher biohydrogenation, and higher deposition of SFA. Furthermore, the incorporation of fatty acids synthesized in muscle tissue may have been more effective in animals subjected to AL intake and 30% FR. This is related to a more lipogenic substrate for *de novo* synthesis in muscle adipocytes [58, 59], especially glucose from the propionate originating from the fermentation of carbohydrates in the rumen [60].

In our study, the lowest IMF deposit was in the meat of animals subjected to 60% FR, which justified the lower concentration of CLA in meat, as CLA is preferentially deposited in triglycerides [61, 62]. Similarly, the lowest IMF deposits in the meat of animals subjected to 60% FR may explain the higher concentration of long chain PUFAs (EPA and AA) in meat, which are deposited primarily in phospholipids [63, 64]. The IMF consists of triglycerides deposited in adipocytes and myofibril cytoplasm droplets, structural phospholipids and cholesterol present in membranes [58]. Triglycerides are more mobile, and phospholipids are more stable in muscle [65].

Based on the results of this study, lambs subjected to 60% FR can be expected to produce meat with fatty acid concentrations that are less favorable to consumer health and with a lower amount of desirable fatty acids, a lower h/H ratio, a higher AI and TI and a higher proportion of SFA (55.3%) compared to those of animals submitted to AL intake (48.3%) and 30% FR (49.2%). A lipid profile favorable to the thrombogenicity and atherogenicity in the meat of animals subjected to 60% FR is related to myristic acid, palmitic acid and stearic acid concentrations [24]. Values from 0.9 to 1.94 for TI and 0.59 to 1.15 for AI have been reported in the literature [66–69]; the maximum values of TI (1.93) and AI (0.87) found in this study were within this range.

The activity of the elongase enzyme is related to concentrations of palmitic, palmitoleic and oleic acids [11, 70]. Combined concentrations of these fatty acids resulted in increased activity of elongase in the muscle of castrated male animals. The lower activity of the Δ9 desaturase enzyme C18 in animals subjected to 60% FR could be attributed to lower amounts of oleic acid present in the muscle of these animals [71].

## Conclusions

Lambs in different sexes produced carcasses with different characteristics, and except for lightness, sex did not influence meat quality or chemical composition. However, females had a fatty acid profile in their meat that was less favorable to consumer health. FR affected carcass traits without influencing the quality of the meat. IMF content decreased when animals were subjected to 60% FR, but the lipid profile was less favorable to consumer health.

## Abbreviations

a*: Redness
ADF: Acid detergent fiber
ADG: Average daily gain
AI: Atherogenicity index
AL: Ad libitum
b*: Yellowness
BW: Body weight
BWS: Body weight at slaughter
C*: Chroma
CCW: Cold carcass weight
CCY: Cold carcass yield
CP: Crude protein
CWL: Cooking weight losses
DFA: Desirable fatty acids
DM: Dry matter
DMI: Dry matter intake
EBW: Empty body weight
EE: Ether extract
FR: Feed restriction
HCW: Hot carcass weight
HCY: Hot carcass yield
IBW: Initial body weight
L*: Lightness
*LL*: *longissimus lumborum*
*LT*: *longissimus thoracis*
MDA: Malondialdehyde
MUFA: Monounsaturated fatty acid
NDF: Neutral detergent fiber
NFC: Non-fiber carbohydrate
NRC: National research council
PUFA: Polyunsaturated fatty acid
REA: Rib eye area
SAS: Statistical analysis software
SFA: Saturated fatty acid
SFT: Subcutaneous fat thickness
TBARS: 2-thiobarbituric acid reactive substances
TI: Thrombogenicity index
TWG: Total weight gain
UFA: Unsaturated fatty acid
WHC: Water holding capacity
ω3: Omega 3
ω6: Omega 6
